# Detection of Zak phases and topological invariants in a chiral quantum walk of twisted photons

**DOI:** 10.1038/ncomms15516

**Published:** 2017-06-01

**Authors:** Filippo Cardano, Alessio D’Errico, Alexandre Dauphin, Maria Maffei, Bruno Piccirillo, Corrado de Lisio, Giulio De Filippis, Vittorio Cataudella, Enrico Santamato, Lorenzo Marrucci, Maciej Lewenstein, Pietro Massignan

**Affiliations:** 1 Dipartimento di Fisica, Università di Napoli Federico II, Complesso Universitario di Monte Sant’Angelo, Via Cintia, Napoli 80126, Italy; 2 ICFO-Institut de Ciencies Fotoniques, The Barcelona Institute of Science and Technology, Av. Carl Friedrich Gauss 3, Castelldefels 08860, Spain; 3 CNR-SPIN, Complesso Universitario di Monte Sant’Angelo, Via Cintia, Napoli 80126, Italy; 4 CNR-ISASI, Institute of Applied Science and Intelligent Systems, Via Campi Flegrei 34, Pozzuoli (NA) 80078, Italy; 5 ICREA—Institució Catalana de Recerca i Estudis Avançats, Pg. Lluis Companys 23, Barcelona E-08010, Spain

## Abstract

Topological insulators are fascinating states of matter exhibiting protected edge states and robust quantized features in their bulk. Here we propose and validate experimentally a method to detect topological properties in the bulk of one-dimensional chiral systems. We first introduce the mean chiral displacement, an observable that rapidly approaches a value proportional to the Zak phase during the free evolution of the system. Then we measure the Zak phase in a photonic quantum walk of twisted photons, by observing the mean chiral displacement in its bulk. Next, we measure the Zak phase in an alternative, inequivalent timeframe and combine the two windings to characterize the full phase diagram of this Floquet system. Finally, we prove the robustness of the measure by introducing dynamical disorder in the system. This detection method is extremely general and readily applicable to all present one-dimensional platforms simulating static or Floquet chiral systems.

## Abstract (web summary)

The detection of topological invariants in the bulk remains challenging even in state-of-the-art experiments. Here, Cardano *et al*. propose a method to read-out the Zak phases and topological invariants in one-dimensional chiral systems and detect those in a photonic quantum walk of twisted photons.

Topological phases of matter escape the canonical characterization of states dictated by the Ginzburg–Landau theory of phase transitions. These phases emerge without breaking symmetries and are not characterized by a long-range order nor a local order parameter but rather by a global topological order. Historically, topology was first proven to have a key role in explaining algebraically decaying order, transport and coherence of two-dimensional Bose liquids, XY models and crystals^1^. Shortly after, the quantization of Hall conductance^2^ was shown to be rooted in current-carrying edge states, protected by the topology of the bulk^3^,^4^,^5^. Being associated with a global order, these phases are robust against local perturbations and promise important applications in metrology, spintronics and quantum computation (see, for example, refs 6, 7, 8).

Intense studies^9^ followed the early discoveries, and topological insulators have by now been engineered in a variety of physical architectures, such as superconducting^10^, mechanical^11^, optomechanical^12^, photonic^13^, atomic^14^ and acoustic platforms^15^. Such diverse systems have been exposed to either real or synthetic magnetic fields, and their topological properties have been studied by scattering at the interface between different domains^15^,^16^ or imaging edge states^17^,^18^,^19^,^20^,^21^,^22^,^23^,^24^,^25^,^26^. Direct detection of topological invariants in the bulk of the system (with no need of edges) has been reported so far by very few experiments^27^,^28^,^29^.

Topological insulators are classified in terms of dimensionality and discrete symmetries^30^. One-dimensional (1D) systems with chiral symmetry are characterized by the Zak phase, that is, the Berry phase accumulated by an eigenstate during its parallel transport through the whole Brillouin zone^31^. The Zak phase is closely related to the electric polarization in solids and plays a key role in the modern theory of insulators^32^,^33^.

Periodically driven (Floquet) systems are attracting an increasing interest, as these show richer topological features than their static counterparts^17^,^24^,^25^,^26^,^34^,^35^,^36^,^37^,^38^,^39^,^40^,^41^,^42^,^43^,^44^. Particularly promising Floquet topological systems are discrete-time quantum walks (QWs)^16^,^17^,^29^,^45^,^46^,^47^, and recent works have reported the observation of topological invariants^16^,^29^, quantum phase transitions^46^ and edge states^17^ in these systems. In its simplest version, a QW is the discrete time evolution of a particle (the walker) on a 1D lattice^48^. At each step, the walker moves to neighbouring sites, with the direction of the shift depending on the state of an internal two-level degree of freedom (the coin). Between consecutive steps, a rotation modifies the coin state, univoquely determining the following evolution.

Here we demonstrate that, in chiral 1D static and Floquet systems with spin 1/2 (that is, a two-state coin), the mean chiral displacement of a particle’s wavepacket becomes quantized and proportional to the Zak phase in the long-time limit. Remarkably, this occurs during the free evolution of the system, in absence of any external force or loss mechanism, with the only requirement that the initial wavefunction is localized. We validate experimentally this finding in a photonic discrete-time QW based on the orbital angular momentum (OAM) of a light beam. We implement the same QW in a shifted inequivalent timeframe and measure a second Zak phase. Combining the two windings, we extract the complete set of topological invariants characterizing the system. Finally, we prove the robustness of our detection by adding dynamical disorder. These measurements provide therefore a bulk measurement of the Zak phases and complete topological invariants of a 1D chiral QW. Our proposal may be straightforwardly applied to general driven Floquet systems.

## Results

### Zak phase detection in the bulk of a QW

**Figure 1:**
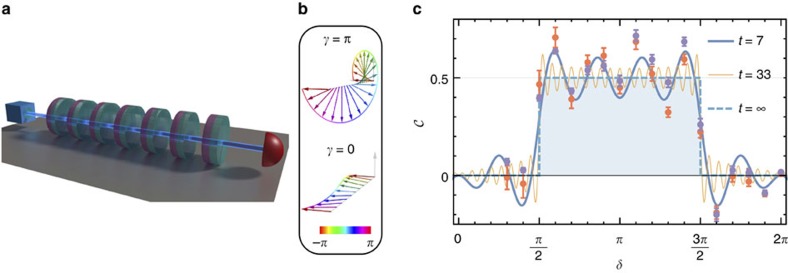
Zak phase detection through the mean chiral displacement. (**a**) Sketch of the setup implementing the protocol *U*=*Q*·*W*. A light beam, exiting a single-mode fibre depicted on the left, performs a QW by propagating through a sequence of quarter-wave plates (purple disks) and *q*-plates (turquoise disks). (**b**) The unit vector **n**(*k*) winds either 1 or 0 times around the chiral axis, as *k* traverses the whole Brillouin zone, depending on the value of the optical retardation *δ*. (**c**) Mean chiral displacement 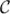 after a 7-step QW of protocol *U*, versus the optical retardation *δ*. Each datapoint is an average over 10 different measurements (error bars are the associated s.e.). Purple and red dots refer, respectively, to different input polarizations, 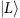 and 
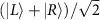
. The lines represent the function *S*_Γ_(*t*) given in equation (7), for different values of the time *t*. In the long-time limit, *S*_Γ_(*t*) converges to (a multiple of) the Zak phase *γ* of protocol *U*.

In one dimension, discrete-time QWs with chiral symmetry display a quantized Zak phase and have been extensively studied in the past years. Among these implementations, we focus on the photonic platform proposed in ref. 46. Here the walk takes place on a lattice whose sites 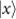 are associated with photonics states 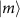, corresponding to light beams that carry *mħ* units of OAM per photon along the propagation axis and show a twisted wavefront^49^. The two coin states are instead mapped onto the left and right circular polarizations of the beam, carrying ±*ħ* units of spin angular momentum per photon along the propagation axis. Once the system is prepared in an initial state 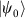, its state after *t* timesteps is given by


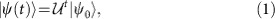


where the single-step operator 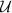 is obtained by cascading suitable combinations of quarter-wave plates and *q*-plates^46^,^50^,^51^. In Fig. 1a, we show a pictorial representation of our setup that realizes a seven-step QW with 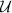 implemented specifically as *U* ≡ *Q*·*W*^46^. The action of a quarter-wave plate oriented at 90° with respect to the horizontal direction is described by the local operator *W*, rotating the polarization states as


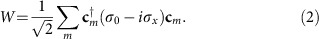


Here 
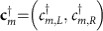
 creates a particle on site *m* with polarization *L*/*R* and *σ*_*i*_ are Pauli matrices acting in the coin (polarization) space. The translation operator *Q* is implemented by a *q*-plate, a liquid crystal device that yields an effective spin–orbit interaction in the light beam. This couples neighbouring sites and polarization states as


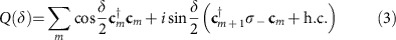


where 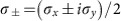 are the operators that flip the coin states 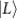 and 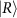, *δ* is the optical retardation of the *q*-plate and h.c. stands for Hermitian conjugate. Further details on the *q*-plates and on the complete experimental setup are provided in the Methods section and Supplementary Fig. 1.

Very generally, QWs are generated by the repeated application of a unitary operator 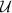, and therefore the system can be described in the framework of Floquet theory. As a consequence of translational invariance in space, the effective Hamiltonian associated with a full period is diagonal in momentum space and may be written as


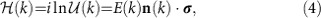


with *E*(*k*) the quasi-energy dispersion, 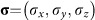 and we have set the period *T* and *ħ* to unity. The point on the Bloch sphere identified by the unit vector **n**(*k*) represents the coin part of the system eigenstates, while their spatial part is a plane wave with quasi-momentum *k*^46^. The function ln(*x*) denotes the principal branch of the natural (matrix) logarithm, so that the quasi-energy is a periodic function, with −*π* and +*π* identified.

The class of QWs we are considering features chiral symmetry, since there exists a unitary operator Γ such that Γ^2^=*I* and 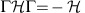. These conditions imply that Γ is Hermitian and that 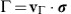, with **v**_Γ_ a vector labelling a point on the Bloch sphere. In this case, the unit vector **n** is bound to rotate around the origin in a plane orthogonal to **v**_Γ_, and the Zak phase equals


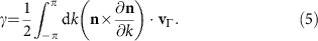


The winding number *γ*/*π* assumes strictly integer values and counts the number of times the unit vector **n** rotates around the unit vector **v**_Γ_ as *k* traverses the whole Brillouin zone. In Fig. 1b, we show the winding of the vector **n** of the operator *U*, for two values of *δ* in different topological sectors. The Zak phase is therefore a bulk property; although it has strong influences in properties of systems where it arises, its detection in current experimental architectures remains challenging.

In the following, we show that information on such topological invariant is hidden in the subleading terms of the mean displacement 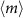, when the initial wavepacket is localized on a single site. This extends a previous result showing that, in the same conditions, the ballistic terms of higher moments of the walker’s displacement feature discontinuities at topological phase transitions^46^. Let us consider the evolution of a wavepacket 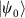 initially localized at site *m*=0, and whose polarization is characterized by the expectation values of the three Pauli matrices, 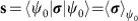. The mean displacement of the wavepacket after *t* timesteps is given by (see Supplementary Note 1 for details)


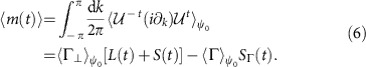


The term in square brackets in equation (6) is proportional to 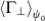, the projection of the initial polarization on a direction orthogonal to **v**_Γ_, and contains a ballistic term *L*(*t*) (which grows linearly with *t*) and a subleading part *S*(*t*).

The vector identifying the specific direction of 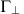 in the plane orthogonal to **v**_Γ_, and the explicit functional forms of *L*(*t*) and *S*(*t*), are non-universal features that depend on the specific protocol (or timeframe) and have no particular relevance for our discussion. The second term in equation (6), which is weighted by 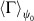 (the projection of the initial polarization along **v**_Γ_), is the subleading chiral term *S*_Γ_ that may be written as (see Supplementary Note 1 for details)


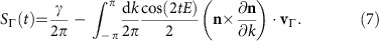


In the limit *t*→∞, *S*_Γ_ becomes proportional to the Zak phase, as the oscillatory correction quickly averages to zero (see Fig. 1c).

The above analysis shows that information on the Zak phase is contained in the mean displacement of the walker, and it may be extracted by fitting 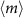 at long times, isolating in turn the second term of equation (6). A related result for the case of a non-Hermitian QW initialized on a chiral eigenstate (that is, an initial condition such that 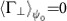) was demonstrated theoretically in ref. 52 and verified experimentally in ref. 29. However, this measurement would not be robust. Indeed, even if one prepared the initial polarization in an eigenstate of the chiral operator Γ, so that 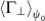
=0, disorder during the propagation of the beam would introduce polarization components orthogonal to **v**_Γ_. These would give rise to ballistic contributions, which in the long-time limit would dramatically affect the result.

An alternative and more convenient approach consists in measuring the mean chiral displacement


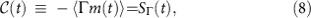


which quantifies the relative shift between the two projections of the state onto the eigenstates of the chiral operator (see Supplementary Note 1 for a concise derivation of this equality). Importantly, the result contained in equation (8) is (i) independent of the initial polarization and (ii) robust against disorder. We probe the chiral displacement in our photonic platform by performing a seven-step QW of the protocol *U*=*Q*·*W*, as depicted in Fig. 1a. The chiral eigenstates correspond to two specific orthogonal polarization states, which depend explicitly on the protocol, and which we detect at the end of the QW (see Methods section). In Fig. 1c, we report the measured values of 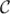 for two different initial polarization states. Experimental points closely follow the theory curve for seven time steps (blue solid line), and no significant differences can be observed between the two different initial states, proving that this measurement is insensitive to the choice of the polarization of the photons. For completeness, we also show results predicted for 33 steps, and the asymptotic long-time limit, which coincides with the Zak phase (over 2*π*). We note here that, although both theory and data oscillate, as few as seven steps are enough to have a clear detection of the Zak phase.

### Zak phase in a shifted timeframe

In static systems, bulk topological invariants such as the Zak phase or the Chern number are uniquely defined by integrals over the whole Brillouin zone and are in one-to-one correspondence with the presence of edge states, thus providing a full classification in terms of the periodic table of topological insulators^30^. The situation is very different in periodically driven (Floquet) systems in *D* dimensions, where the integral determining the topological invariants needs to be performed over a *D*+1 dimensional torus constituted by the Brillouin zone and an extra periodic dimension, the quasi-energy^42^.

**Figure 2:**
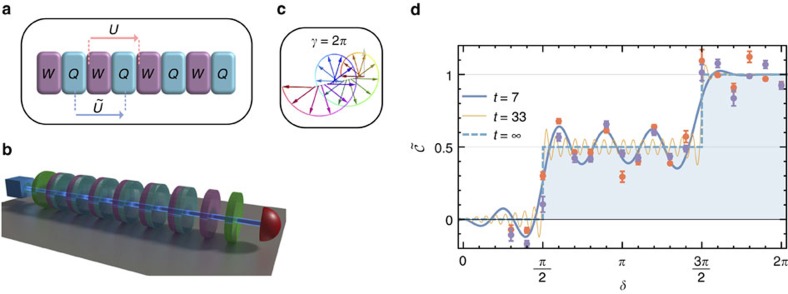
Zak phase in the complementary timeframe. (**a**) Different choices of the origin of the periodic cycle lead to different protocols. (**b**) Sketch of the setup implementing protocol 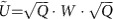. The two *q*-plates at the beginning and end of the optical path (shown in bright green) yield an optical retardation *δ*/2, where *δ* is the optical retardation characterizing bulk *q*-plates (turquoise). (**c**) The unit vector 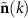 associated with the operator 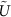, for optical retardations 3*π*/2<*δ*<2*π*, winds twice around the chiral axis as *k* spans the whole Brillouin zone. (**d**) Mean chiral displacement 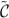 after a 7-step QW with protocol 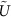. The datapoints are averages of 10 experimental measurements, and error bars are the associated s.e. Purple and orange colours refer, respectively, to input polarizations 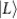 and 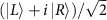. The lines display 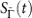, obtained by replacing **n** with 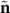 in equation (7), for different values of the time *t*. At long times, 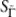 converges to the Zak phase 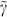.

Moreover, a gauge freedom is introduced by the choice of the timeframe, that is, the origin of time of the periodic cycle (see Fig. 2a). While the dispersion *E*(*k*) is equal in all timeframes, the effective Hamiltonian, its eigenstates and symmetries and the resulting dynamics crucially depend on the timeframe^40^. As an example, the operator 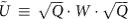 defines a timeframe that is inequivalent to the one introduced by *U*. In particular, the unit vector 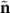
(*k*) defined by 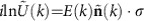 may wind twice around the chiral axis as *k* traverses the Brillouin zone (see Fig. 2c), and its Zak phase 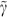 (dashed line in Fig. 2d) differs from the Zak phase *γ* of protocol *U* (dashed line in Fig. 1c).

We realize experimentally protocol 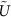 by the setup shown schematically in Fig. 2b. Using the relation 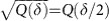, it is straightforward to see that 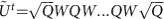. Hence, we realize the operator 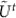 by placing *q*-plates yielding an optical retardation *δ*/2 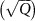 at the beginning and end of the optical path, while in the bulk of the walk we adopt the same sequence reported in Fig. 1a (with the last *q*-plate removed). Overall, our QW implements seven steps of protocol 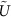 by means of a total of eight *q*-plates, six with retardation *δ* and two tuned at *δ*/2 (first and last plates), separated by quarter-wave plates. In Fig. 2d, we report the measure of the mean chiral displacement 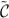 generated by the single-step operator 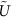. As in the case of protocol *U*, this quantity accurately follows the theory prediction, providing an unambiguous detection of the Zak phase 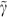 of the infinite system after just seven steps.

### Complete topological characterization

**Figure 3:**
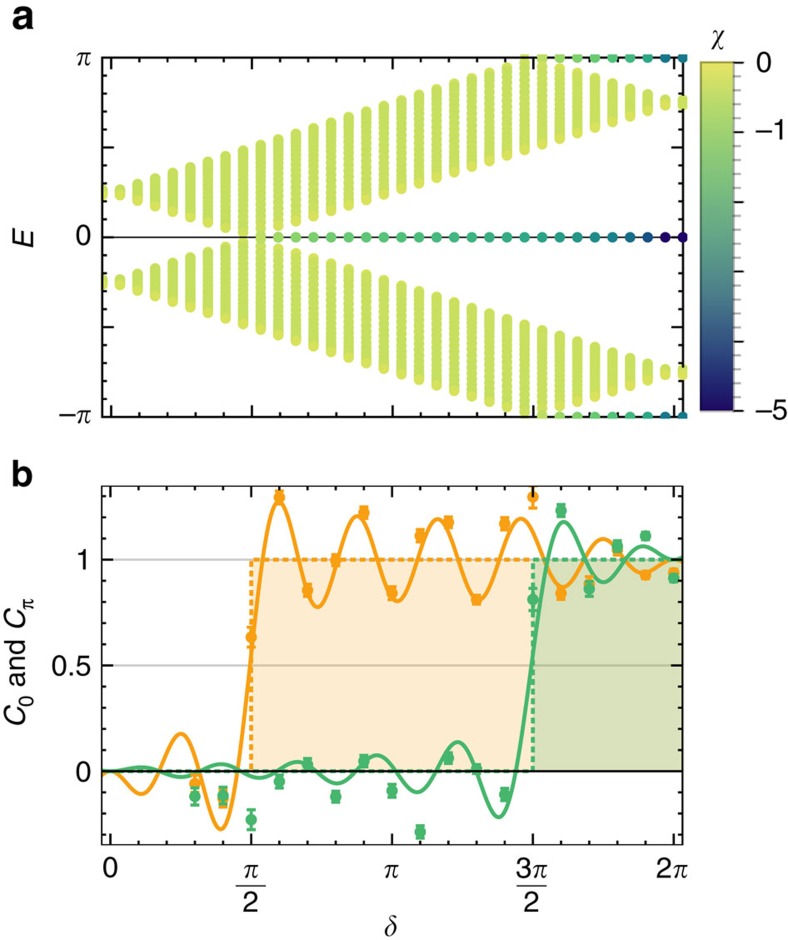
Topological invariants and bulk-edge correspondence. (**a**) Edge states on an open-ended lattice [−*L* : *L*], with *L*=10; the colour coding indicates the degree of localization 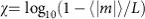, with darker colours indicating states more localized towards the edges. (**b**) Topological invariants *C*_0_ and *C*_*π*_, obtained as in equation (9) by combining the measurements of the mean chiral displacements 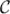 and 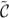 of protocols *U* and 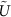, and averaging the results obtained from the two initial states (error bars are the propagated s.e.). The dashed lines show the long-time limit of the topological indices *C*_0_ and *C*_*π*_, yielding respectively the number of edge states at 0 and *π* energy.

It is clear from the previous discussion that the Zak phase associated with a single timeframe does not contain all the topological information of our QW. Indeed in Floquet 1D chiral systems, there exist two independent classes of protected edge states at either 0- and *π* energies. An example of these edge states is shown in Fig. 3a, where we plot the quasi-energies of all eigenstates of an open-ended lattice. As remarked above, the spectrum is independent of the timeframe. The spectrum contains edge states even for 3*π*/2<*δ*<5*π*/2 where the Zak phase *γ* of protocol *U* is zero, explicitly confirming that the Zak phase of a single QW protocol does not contain the complete information about the topological state of the system.

The bulk-edge correspondence in these driven systems requires two invariants *C*_0_ and *C*_*π*_, yielding, respectively, the number of 0- and *π*-energy edge states. As shown in refs 38, 41, these are simple functions of two Zak phases, measured in two inequivalent timeframes possessing an ‘inversion point’, that is, which may be written, respectively, as 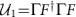 and 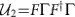, with *F* a suitable evolution operator. In the case of our setup, the two special protocols fulfilling this criterion are 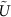 and 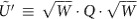. However, it is simple to show that 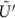 is topologically equivalent to *U*, as no gap closing happens during the rotation 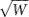; therefore the Zak phase of 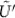 coincides with *γ*. As such, the complete topological classification of 1D chiral systems may be obtained by means of the two quantities


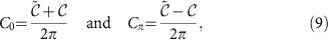


which converge in the long-time limit, respectively, to the number of 0- and *π*-energy edge states. By combining our measurements of the mean chiral displacements measured in the inequivalent timeframes, we are now able to compute the invariants *C*_0_ and *C*_*π*_ and detect the complete phase diagram of this system: the result is shown in Fig. 3b. Once again, our measurements show a remarkably fast convergence towards the asymptotic limit.

### Robustness to dynamical disorder

Finally, we test the stability of the quantization of the mean chiral displacement against disorder. In particular, we choose protocol *U* and introduce dynamical disorder by offsetting the optical retardation *δ*_*j*_ (1≤*j*≤7) of each *q*-plate by a small random amount 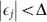 around their common mean value 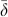. In our experiment, we set Δ=*π*/10 and *π*/5. We note that this disorder is dynamic, in the sense that it affects independently the various *q*-plates crossed by the beam, but crucially it respects chiral symmetry. This can be simply understood by noting that the vector **v**_Γ_, defining the chiral operator, does not depend on *δ*.

**Figure 4:**
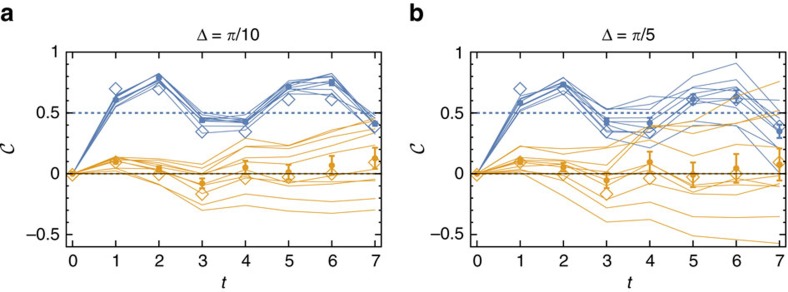
Robustness to dynamical disorder. Measurement of the mean chiral displacement 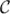 of protocol *U* for a localized input state in the presence of dynamical disorder. For the orange (blue) lines, we choose a mean value of the *q*-plate optical retardation 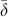
=7*π*/4 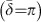, expected to yield a Zak phase of *γ*/2*π*=0 (*γ*/2*π*=1/2), and we add at each time step a small random retardation 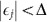, with Δ=*π*/10 (**a**) and *π*/5 (**b**). Thin solid lines display the measurements of single realizations, and their average is shown as filled circles (error bars are the s.e.m.). In all plots, empty diamonds represent theoretical simulation calculated for the ideal case Δ=0, and dotted lines the expected result for *t*→∞.

As shown in Fig. 4, in single realizations the mean chiral displacement presents oscillations featuring higher amplitude for increasing disorder, but an ensemble average over independent realizations smoothly converges to the expected theoretical result, which in the infinite time limit gives the bulk value of the Zak phase. Here we performed measurements on protocol *U*, but similar robustness of the chiral displacement shall hold for every 1D QW chiral protocol, and more generally every 1D chiral system, as long of course as the disorder does not break chiral symmetry and its strength is small compared to the gap size to prevent interband transitions. As an example, in the Supplementary Note 2 and Supplementary Figs 2–5 we show that the mean chiral displacement is an equally robust topological marker for a completely different and static (that is, not driven) system, the celebrated SSH model.

## Discussion

Summarizing, here we proposed an efficient method to measure the Zak phase of a chiral system by direct observation of its free bulk dynamics. In particular, we showed that information on the topological phase of the bulk is encoded in the mean chiral displacement, an oscillatory quantity that rapidly converges to the Zak phase, and is robust against (chiral-preserving) disorder in both space and time.

We experimentally verified our findings by performing the first measurement of the Zak phase of a chiral QW. The physical platform we chose is a photonic setup based on the OAM of a light beam, where the mean chiral displacement corresponds to the relative shift of the two chiral polarization components. A precise readout of the Zak phase was obtained after only seven QW steps. We further used the same method to measure the Zak phase in a complementary timeframe, which we realized by swapping few optical components. By combining the two measurements, we extracted the two invariants providing the complete bulk-edge correspondence for this driven system, that is, the one associated to the 0-energy edge state, and the one connected to the anomalous *π*-energy edge state. Finally, we proved that the mean chiral displacement is a robust measure of the Zak phase by introducing dynamical but chiral-preserving disorder.

Although here we investigated experimentally a specific QW, our results are not restricted to QWs nor to Floquet systems. Indeed, the mean chiral displacement provides a robust topological characterization of arbitrary spin-1/2 1D chiral systems, either static or periodically driven. These may nowadays be realized in a variety of platforms, ranging from ultracold atoms in optical lattices to photonic waveguides and from semiconductor quantum wells to optomechanical systems.

While formerly known methods for detection of topological properties require a uniform filling of the band of interest, external forces, loss mechanisms or fine-tuning so that only edge states are populated, the method proposed here quite remarkably achieves this goal by observing the free evolution of a single particle, initially localized on a single site in the bulk. This aspect may be specially beneficial for systems where filling a band is intrinsically challenging, such as bosonic condensates or phononic and photonic ensembles.

Future interesting directions opened by this work include the extension of our results to chiral systems with more than two internal states, a further understanding of the role played by temporal disorder and the topological characterization of systems in higher spatial dimensions.

## Methods

### Experimental setup

Our apparatus is shown schematically in Figs 1a and 2b, and a more detailed description is given in Supplementary Fig. 1. We produce a TEM_00_ mode by coupling the output of a Ti:Sa laser (*λ*=800 nm) to a single-mode fibre, thus preparing the beam in an OAM state with *m*=0. At the exit of the fibre, a specific polarization is selected by means of a sequence of a quarter-wave plate and a half-wave plate. Therefore, the initial state of the QW is 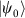
=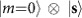, where *m* is the position in the walker (OAM) and space ***s*** its coin state (polarization). In the standard protocol *U*=*Q*·*W*, the single step consists of a quarter-wave plate oriented at 90° with respect to the horizontal direction (operator *W*), followed by a *q*-plate (operator *Q*), as shown in Fig. 1a. To implement the second protocol, we exploited the fact that equation (3) may be written as 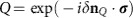, that is, it corresponds to a rotation around a suitable unit vector **n**_*Q*_; as such, a *q*-plate with retardation *δ*/2 implements the desired operator 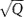. To implement the single-step operator 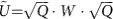, we then added a *δ*/2 *q*-plate at the beginning of the sequence, and we halved the retardation of the last *q*-plate, as shown in Fig. 2b.

### *q*-plates

Each *q*-plate consists of a thin layer of birefringent liquid crystals, whose optic axes are arranged in a singular pattern characterized by a topological charge *q* (in our case, *q*=1/2). The patterned birefringence gives rise to an optical spin–orbit coupling that induces the polarization-dependent shift of OAM. Along with the specific pattern, the action of each device is determined by its optical retardation *δ*, as reported in equation (3). The optical retardation can be continuously tuned by applying an electric field, allowing in turn for an accurate control of the spin–orbit interaction^53^.

### Detection of the chiral displacement

At the end of the walk, we can select any polarization component of the final state by a combination of a quarter-wave and a half-wave plate, followed by a linear polarizer, and we measure its OAM content by diffraction on a spatial light modulator coupled to a single-mode fibre and a power meter, which records the light intensity. Since we are interested in analysing the OAM spectrum of chiral components of the final wavepacket, waveplates orientations are selected so as to implement polarization projections onto chiral states 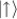 and 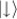. The chiral operators for protocols *U* and 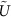 are, respectively, 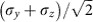 and *σ*_*z*_, so it is straightforward to see that 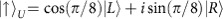 and 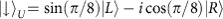 for protocol *U*, while 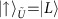 and 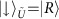 for protocol 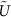. The combination of polarization and OAM projections allows for determining the probabilities *P*_*i*,*m*_, with *i*={↑, ↓}, that the system is in the chiral state 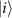 and in the OAM state 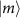. Given the probability distributions *P*_*i*,*m*_, the chiral displacement is simply given by 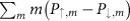.

### Data availability

The complete set of raw data supporting the findings of this study is available from the corresponding authors upon request.

## Additional information

**How to cite this article:** Cardano, F. *et al*. Detection of Zak phases and topological invariants in a chiral quantum walk of twisted photons. *Nat. Commun.* **8**, 15516 doi: 10.1038/ncomms15516 (2017).

**Publisher’s note**: Springer Nature remains neutral with regard to jurisdictional claims in published maps and institutional affiliations.

## Supplementary Material

Supplementary Information Supplementary Figures and Supplementary Notes

Peer Review File
