# Supplementary material for: Detection of Zak phases and topological invariants in a chiral quantum walk of twisted photons
Source: Nat Commun. 2017 Jun 1;8:15516. doi: 10.1038/ncomms15516 (PMC5501976; doi:10.1038/ncomms15516)
Supplement: Supplementary Information — Supplementary Figures and Supplementary Notes [file ncomms15516-s1.pdf]

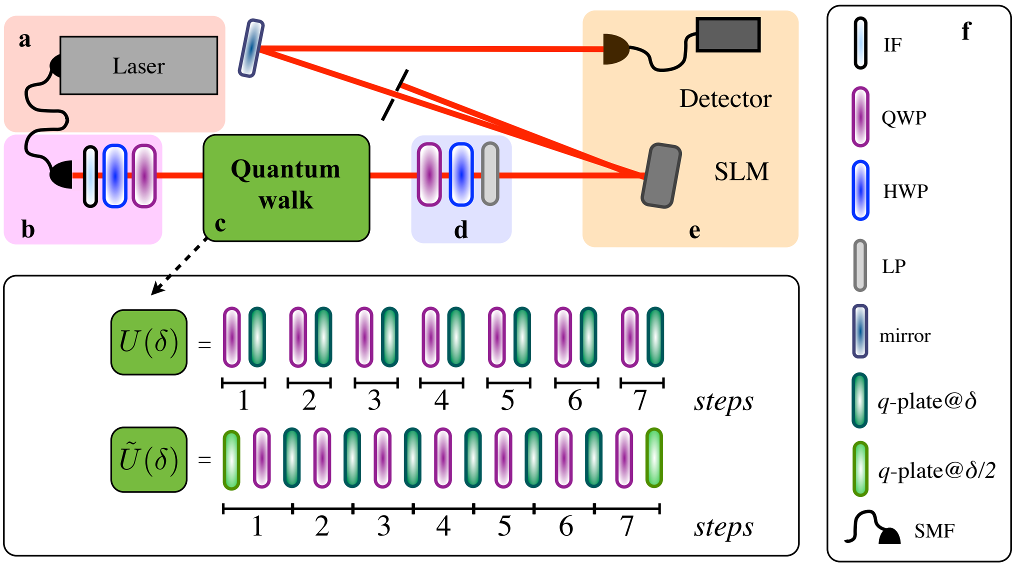

Supplementary Figure 1. **Scheme of the experimental setup**. **(a)** The output of a Ti:Sa pulsed laser source (pulse duration = 100 fs, central wavelength = 800 nm, repetition rate = 82 MHz) is coupled into a single mode fiber (SMF) so as to clean the laser spatial mode; this provides a single OAM state with $m = 0$ at the input of the QW. **(b)** At the exit of the fiber, the beam passes through an interferential filter (IF), whose transmittance is peaked at 800 nm with bandwidth of 3 nm, which allows to have a stable control of the light's wavelength and a narrower frequency distribution. Then the desired input polarization state is prepared by means of a half-wave plate (HWP) and a quarter-wave plate (QWP). **(c)** The light beam passes through a sequence of QWPs and q-plates, as shown in detail in the inset, which are positioned in order to realize either protocol U or $\tilde{U}$. **(d)** At the end of the QW, a polarization component is selected by means of a QWP and a HWP, followed by a linear polarizer (LP). **(e)** The OAM spectrum is measured by diffraction on a spatial light modulator (SLM), that displays standard pitchfork holograms for the projection over OAM states. At the first diffraction order, the light is coupled into a SMF that is directly connected to a power meter recording the field intensity. **(f)** Legend of optical components displayed in panels **(a-e)**.

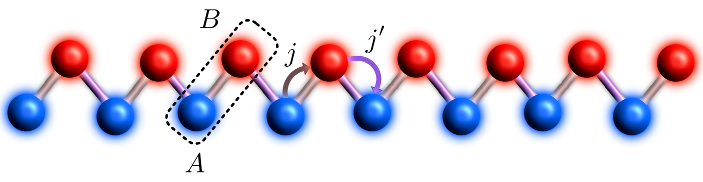

Supplementary Figure 2. **SSH model.** The SSH model describes a one-dimensional lattice with each unit cell containing two sites, A (blue) and B (red). The Hamiltonian has an intra-cell hopping of amplitude j and an inter-cell hopping of amplitude j'.

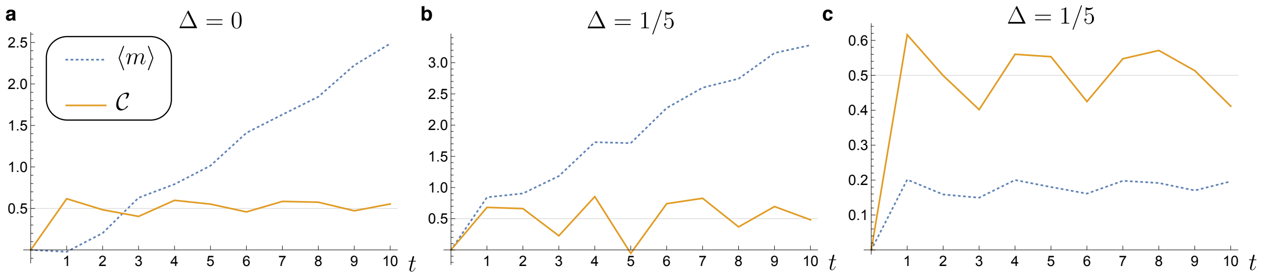

Supplementary Figure 3. **Disordered SSH model.** Evolution of a walker on an SSH lattice with dynamical disorder in the tunnelings. At $t = 0$, the walker is initialized on the central unit cell of the chain, with a random polarization (different for each realization), and we have taken $j = j'/2$, so that the model has a Zak phase $\gamma = \pi$. Dashed (solid) lines depict the mean (mean chiral) displacement. **(a)** Single realization in absence of disorder. **(b)** Single realization with disorder amplitude $\Delta = 1/5$. **(c)** Upon ensemble-averaging over 100 realizations (with $\Delta = 1/5$), the mean chiral displacement smoothly converges to $\gamma/(2\pi)$.

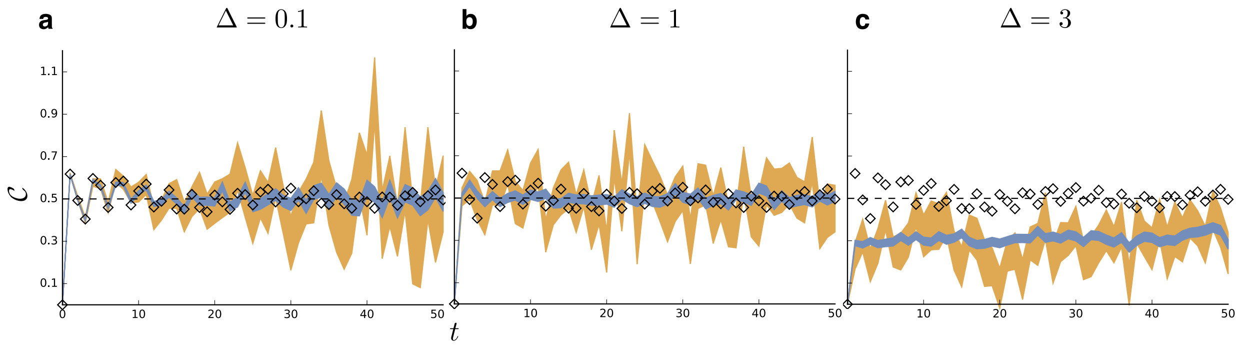

Supplementary Figure 4. **Long-time behavior with static disorder.** Mean chiral displacement $\mathcal{C}$ of the SSH model with $j' = 2j$ and static disorder over all tunnelings, of amplitude **(a)** $\Delta = 0.1$,**(b)** $\Delta = 1$ and **(c)** $\Delta = 3$. The yellow (blue) line indicates the ensemble average of $\mathcal{C}$ over 50 (1000) realizations, and the width of the line corresponds to the standard error of the mean. The black diamonds show the result in absence of disorder, where the spectrum has a gap of amplitude $\Delta_{\text{gap}} = 2$. The images show how, in the long time limit, the ensemble average of the mean chiral displacement remains locked to a value consistent with the Zak phase, as long as $\Delta \lesssim \Delta_{\text{gap}}$.

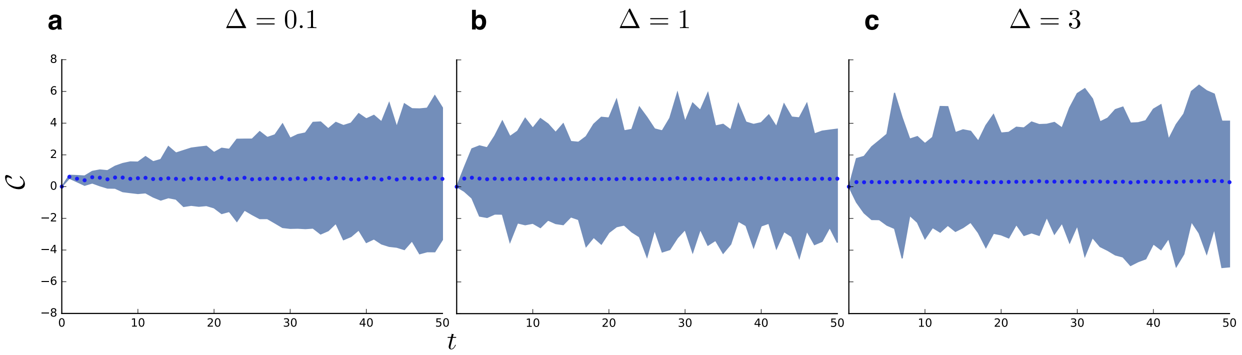

Supplementary Figure 5. **Effect of the disorder on the error in time.** The mean chiral displacement is plotted for each disorder realization (blue area) and is compared to the ensemble average (dots) for **(a)** $\Delta = 0.1$,**(b)** $\Delta = 1$ and **(c)** $\Delta = 3$. For small disorder, the error on the ensemble average grows in time. For important disorder, the error on the ensemble average is independent of the time.

SUPPLEMENTARY NOTE 1: MEAN DISPLACEMENTS

We provide here the detailed derivation of the two key formulas introduced in the text, which yield the mean displacement of the whole wavepacket, and its mean chiral displacement. To start, consider the evolution of a wavepacket $|\psi_0\rangle$ initially localized at site $m=0$, and let its polarization be characterized by the expectation values of the three Pauli operators $\mathbf{s}=\langle\boldsymbol{\sigma}\rangle_{\psi_0}\equiv\langle\psi_0|\boldsymbol{\sigma}|\psi_0\rangle$. The evolution operator generating t timesteps of protocol U is $U^t=(Q.W)^t=\mathrm{e}^{-iEt\mathbf{n}\cdot\boldsymbol{\sigma}}=\cos(Et)\sigma_0-i\sin(Et)\mathbf{n}\cdot\boldsymbol{\sigma}$. Using of the standard identity $(\mathbf{a}\cdot\boldsymbol{\sigma})(\mathbf{b}\cdot\boldsymbol{\sigma})=(\mathbf{a}\cdot\mathbf{b})\sigma_0+i(\mathbf{a}\times\mathbf{b})\boldsymbol{\sigma}$ (valid for arbitrary vectors $\mathbf{a}$ and $\mathbf{b}$), it is straightforward to show that the mean displacement of the wavepacket reads:

$$\begin{aligned}\langle m(t)\rangle=\langle\psi(t)|m|\psi(t)\rangle&=\int_{-\pi}^{\pi}\frac{\mathrm{d}k}{2\pi}\left\langle U^{-t}(i\partial_k)U^t\right\rangle_{\psi_0}\\&=\int_{-\pi}^{\pi}\frac{\mathrm{d}k}{2\pi}\langle[\cos(Et)\sigma_0+i\sin(Et)\mathbf{n}\cdot\boldsymbol{\sigma}]\,(i\partial_k)\,[\cos(Et)\sigma_0-i\sin(Et)\mathbf{n}\cdot\boldsymbol{\sigma}]\rangle_{\psi_0}\\&=\int_{-\pi}^{\pi}\frac{\mathrm{d}k}{2\pi}\left[\left(t\frac{\partial E}{\partial k}-\frac{\partial}{\partial k}\frac{\sin(2Et)}{2}\right)\mathbf{n}-\sin(Et)^2\left(\mathbf{n}\times\frac{\partial\mathbf{n}}{\partial k}\right)\right]\cdot\mathbf{s}\,.\end{aligned}\tag{1}$$

When a protocol possesses chiral symmetry (and both ours do), the unit vector $\mathbf{n}$ of the corresponding effective Hamiltonian is bound to rotate in the plane orthogonal to the vector $\mathbf{v}_\Gamma=\mathrm{tr}(\Gamma\boldsymbol{\sigma})/2$ associated to its chiral operator Γ. In turn, this means that $(\mathbf{n}\times\partial_k\mathbf{n})$ is parallel to $\mathbf{v}_\Gamma$. We arrive then to the expression quoted in the text,

$$\langle m(t)\rangle=\langle\Gamma_\perp\rangle_{\psi_0}[L(t)+S(t)]-\langle\Gamma\rangle_{\psi_0}S_\Gamma(t).\tag{2}$$

Specifically, the chiral operator for protocol U is $\frac{\sigma_y+\sigma_z}{\sqrt{2}}$, so that $\langle\Gamma\rangle_{\psi_0}=\frac{s_y+s_z}{\sqrt{2}}$, and one finds $\langle\Gamma_\perp\rangle_{\psi_0}=\frac{s_y-s_z}{\sqrt{2}}$.

We omit here for simplicity the explicit expressions of the functions $\{E,\,\mathbf{n},\,L,\,S\}$ in terms of the optical retardation δ and the quasi-momentum k, as these are protocol-dependent, rather bulky, and not particularly illuminating. However, it is obvious that E and $\mathbf{n}$ are independent of time, so the term $L(t)$ in Supplementary Eq. (2) is ballistic (i.e., grows linearly with t), while $S(t)$ is oscillatory. The last function in Supplementary Eq. (2) is the one we refer to as *chiral term*,

$$S_\Gamma(t)=\int_{-\pi}^{\pi}\frac{\mathrm{d}k}{2\pi}\sin(Et)^2\left(\mathbf{n}\times\frac{\partial\mathbf{n}}{\partial k}\right)\cdot\mathbf{v}_\Gamma=\frac{\gamma}{2\pi}-\int_{-\pi}^{\pi}\frac{\mathrm{d}k}{2\pi}\frac{\cos(2Et)}{2}\left(\mathbf{n}\times\frac{\partial\mathbf{n}}{\partial k}\right)\cdot\mathbf{v}_\Gamma,\tag{3}$$

which is clearly proportional to the Zak phase $\gamma=\frac{1}{2}\int_{-\pi}^{\pi}\mathrm{d}k(\mathbf{n}\times\partial_k\mathbf{n})\cdot\mathbf{v}_\Gamma$, plus an oscillatory contribution whose amplitude and period generally decay rapidly as $t\to\infty$. A singular point is however $\delta=2\pi$, where $E=3\pi/4$, independent of k, so that $\cos(2Et)$ equals 0 for odd t, and $(-1)^{t/2}$ for even t. This means that $S_\Gamma(t)$ at $\delta=2\pi$ equals exactly $\gamma/2\pi$ for odd values of t, while it equals 0 (γ/π) for even (odd) values of $t/2$. For this reason, in Figs. 1-3 of the main text we have considered quantum walks with an odd number of steps.

The derivation of the mean displacement for protocol $\tilde{U}=\sqrt{Q}\cdot W\cdot\sqrt{Q}$ yields formulas which are identical to Supplementary Eqs. (1)-(3), provided one replaces $\mathbf{n}$ with $\tilde{\mathbf{n}}$ and remembers that the chiral operator is σ_z, so that $\langle\tilde{\Gamma}\rangle_{\psi_0}=s_z$, and $\langle\tilde{\Gamma}_\perp\rangle_{\psi_0}=s_y$.

The mean chiral displacement $\mathcal{C}$, Eq. (8) of the main text, may be computed along the same lines. We show here the calculation for the simplest case of the chiral displacement $\tilde{\mathcal{C}}$ associated to the protocol $\tilde{U}$, whose unit vector $\tilde{\mathbf{n}}$ has a vanishing z-component since $\tilde{\Gamma}=\sigma_z$. One finds:

$$\begin{aligned}\tilde{\mathcal{C}}(t)=\langle\tilde{\Gamma}m(t)\rangle&=\int_{-\pi}^{\pi}\frac{\mathrm{d}k}{2\pi}\left\langle\tilde{U}^{-t}\sigma_z(i\partial_k)\tilde{U}^t\right\rangle_{\psi_0}\\&=\int_{-\pi}^{\pi}\frac{\mathrm{d}k}{2\pi}\left\langle\sin(Et)^2\left[(\tilde{\mathbf{n}}\times\partial_k\tilde{\mathbf{n}})_z\,\sigma_0-\frac{i}{2}\partial_k|\tilde{\mathbf{n}}|^2\sigma_z\right]+i\partial_k\left[\cos(Et)\sin(Et)(\tilde{n}_x\sigma_y-\tilde{n}_y\sigma_x)-\sin(Et)^2\sigma_z\right]\right\rangle_{\psi_0}\\&=\int_{-\pi}^{\pi}\frac{\mathrm{d}k}{2\pi}\sin(Et)^2\left(\tilde{\mathbf{n}}\times\frac{\partial\tilde{\mathbf{n}}}{\partial k}\right)_z=S_{\tilde{\Gamma}}(t).\end{aligned}\tag{4}$$

In the second line of Supplementary Eq. (4), all terms preceded by an imaginary unit i integrate to zero: the first because $\tilde{\mathbf{n}}$ is a vector of unit norm for all k, and the second because it is the integral of a total derivative over a closed path. The final result is purely real, in agreement with the fact that the chiral displacement is the expectation value of an Hermitian operator. The derivation of the chiral displacement $\mathcal{C}$ for protocol $U=Q\cdot W$ is completely analogue, provided the reference frame of the Pauli matrices is suitably rotated.

SUPPLEMENTARY NOTE 2: SSH MODEL

The Su-Schrieffer-Heeger (SSH) model, originally introduced to study polyacetylene chains, is probably the simplest and most studied chiral-symmetric topological system. It describes a one-dimensional dimerized lattice, where each unit cell contains two sites labeled A and B. Further on, we will call them alternatively lattice sub-sites or polarizations. The amplitudes j and j' quantify, respectively, the hopping between adjacent sub-sites belonging to the same cell, and to neighboring ones. The Hamiltonian describing the dynamics along a dimerized lattice with N cells reads:

$$H = j\sum_{n=1}^{N} \mathbf{c}_n^\dagger \sigma_x \mathbf{c}_n + j' \sum_{n=1}^{N-1} \left(\mathbf{c}_{n+1}^\dagger \sigma_+ \mathbf{c}_n + \mathbf{c}_n^\dagger \sigma_- \mathbf{c}_{n+1}\right) \tag{5}$$

Here $\mathbf{c}_n^\dagger = (c_{n,A}^\dagger, c_{n,B}^\dagger)^T$ creates a particle on site A and B of cell n, and σ_i are Pauli matrices acting in the sub-lattice space. The Bloch Hamiltonian is a 2×2 matrix:

$$H(k) = [j + j'\cos(ka)]\,\hat{\sigma}_x + j'\sin(ka)\,\hat{\sigma}_y = E(k)\mathbf{n}(k)\cdot\boldsymbol{\sigma}, \tag{6}$$

where the dispersion reads $E(k) = \pm\sqrt{j^2 + j'^2 + 2jj'\cos(k)}$, the real unit vector $\mathbf{n}(k)$ determines the position of the eigenstates on the Bloch sphere, and a is the lattice constant. Since the Hamiltonian doesn't contain direct couplings between $A-A$ or $B-B$ sites, the vector $\mathbf{n}(k)$ lies in the xy plane, so that the chiral symmetry operator Γ is σ_z. Hamiltonians with $j > j'$ and $j < j'$ and are topologically inequivalent, being the winding number (i.e., the Zak phase γ divided by π) equal to zero and one, respectively.

Equations (6) and (8) of the main text, giving respectively the mean displacement and the mean chiral displacement of an initially localized walker performing a chiral quantum walk, describe equally well the dynamics of a particle on the SSH chain, initially localized on a single unit cell. A and B sites of the SSH lattice may be identified with the $|L\rangle$ and $|R\rangle$ polarization states of the quantum walker.

Exactly as for the QW analyzed in the main text, the mean chiral displacement is a robust marker of the topological phase of the SSH lattice. To illustrate this, we simulate the evolution of a localized electron in a lattice with $j'/j > 1$, so that the Zak phase γ is π. As we show in panel (a) of Supplementary Fig. 3, in absence of disorder the mean value of the chiral displacement oscillates around $\gamma/(2\pi) = 1/2$, and converges to this value in the long time limit. To test the robustness of the measurement, we add disorder to the system, in the form of a random dynamical disturbance to each tunneling; specifically, at each timestep t the $A-B$ tunneling in cell n, $j_{n,t}$, is replaced by $j_{n,t} + \epsilon_{n,t}$, with a randomly chosen $|\epsilon_{n,t}| < \Delta$ (and similarly for the tunneling $j'_{n,t}$ connecting cells n and $n+1$); note that this form of the disorder explicitly preserves the chiral nature of the model. In presence of disorder, like in the QW case discussed in Fig. 4 of the main text, single realizations present oscillations of increasing amplitude with increasing disorder, but an ensemble average over independent realizations smoothly converges to the expected theoretical result; see panels (b) and (c) of Supplementary Fig. 3.

Behavior at very long times. We focus here on the effect of disorder after very long times. In Supplementary Figure 4 we consider the mean chiral displacement $\mathcal{C}$ of the disordered SSH model with $j' = 2j$. The yellow (blue) line displays the ensemble average over 50 (1000) realisations of static disorder with amplitude Δ, while the black diamonds depict the mean chiral displacement in absence of disorder. In the clean case, the system presents an energy gap $\Delta_{\text{gap}} = 2$, and each energy band has a bandwidth of $\Delta_{\text{bw}} = 2$. Supplementary Figure 4a shows the effect for $\Delta = 0.1$: the ensemble average converges to the mean chiral displacement, even in the long time limit.

Supplementary Figure 4b shows the case of $\Delta = 1$, a disorder smaller than the energy gap but comparable to the bandwidth. Strikingly, in this case the ensemble averaged mean chiral displacement converges to the Zak phase faster than in absence of disorder (as may be noticed by comparing the blue line with the black diamonds). This demonstrates that a moderate disorder ($\Delta \lesssim \Delta_{\text{gap}}$) can wash out the coherent oscillations, but it does not destroy topological effects, and actually may even be beneficial, as it provides faster convergence: moderate disorder favors the measure of the topology. Supplementary Figure 4c shows instead the result for a disorder sensibly larger than the energy gap, $\Delta = 3$. In this case, as expected, topological protection is completely lost, and the ensemble averaged mean chiral displacement fails to converge to the Zak phase.

We continue the study of static disorder by commenting on the growth in time of the error on the ensemble average. In Supplementary Figure 5, the blue area denotes the superposition of all 1000 values of $\mathcal{C}$ which were computed to produce Supplementary Figure 4, and the bright blue dots denote their ensemble average. For disorder amplitudes smaller than the bandwidth, we observe that $\mathcal{C}$ undergoes oscillations of steadily growing amplitude in time. Therefore, the longer the time of observation, the more realizations will be needed to minimize the error. When the amplitude of the disorder becomes comparable to the bandwidth, however, $\mathcal{C}$ shows large oscillations even at very short times, but their amplitude does not grow further in time. Therefore, in the long time limit it is not necessary to increase the number of realisations to measure the Zak phase in presence of moderate disorder.
