# Supplementary material for: Detection of Zak phases and topological invariants in a chiral quantum walk of twisted photons
Source: Nat Commun. 2017 Jun 1;8:15516. doi: 10.1038/ncomms15516 (PMC5501976; doi:10.1038/ncomms15516)
Supplement: Peer Review File [file ncomms15516-s2.pdf]

Editorial Note: this manuscript has been previously reviewed at another journal that is not operating a transparent peer review scheme. This document only contains reviewer comments and rebuttal letters for versions considered at Nature Communications.
